# Improving Thermostability of Chimeric Enzymes Generated by Domain Shuffling Between Two Different Original Glucoamylases

**DOI:** 10.3389/fbioe.2022.881421

**Published:** 2022-04-05

**Authors:** Zhongxiu Chen, Longbin Wang, Yuyu Shen, Dunji Hu, Liying Zhou, Fuping Lu, Ming Li

**Affiliations:** ^1^ Key Laboratory of Industrial Fermentation Microbiology (Tianjin University of Science and Technology), Ministry of Education, Tianjin, China; ^2^ College of Biotechnology, Tianjin University of Science and Technology, Tianjin, China; ^3^ Tianjin Key Laboratory of Industrial Microbiology, Tianjin, China

**Keywords:** glucoamylase, domain shuffling, chimeric enzyme, thermostability, enzymatic properties

## Abstract

In order to improve enzymatic properties of glucoamylases, six recombinant genes GA1–GA6 were created by domain shuffling of glucoamylase genes GAA1 from *Aspergillus niger* Ld418AI and GATE from *Talaromyces emersonii* Ld418 TE using overlap extension PCR and were expressed in *Saccharomyces cerevisiae* W303-1B; only activities of GA1 and GA2 in the fermentation broth were higher than those of GAA1 but less than those of GATE. Further research results of GA1 and GA2 indicated that chimeric glucoamylases GA1 and GA2 revealed increased thermostability compared with GAA1 and GATE, although with a slight change in the activity and optimal temperature. However, GA1 had almost the same catalytic efficiency as GATE, whereas the catalytic efficiency of GA2 was slightly less than that of GATE, but still higher than that of GAA1. The structural analysis showed that the change of enzymatic properties could be caused by the increased and extended α-helix and β-sheet, which change the secondary and tertiary structures of chimeric glucoamylases. These results demonstrated that domain shuffling was feasible to generate a chimeric enzyme with novel properties.

## Introduction

Glucoamylase (GA) (α-l, 4-glucan glucohydrolase, EC 3.2.1.3) plays an important role in the fermentation and food industries for saccharification of starch/amylopectin (Kumar and Satyanarayana, 2009; Zong et al., 2022). It can catalyze hydrolysis of α-1,4 glycosidic bonds to release d-glucose residues from the non-reducing ends of starch and related oligo- and polysaccharide chains and also has limited ability to hydrolyze amylopectin α-1,6 linkages resulting in glucose (Sauer et al., 2000). Although glucoamylases can be produced by many fungal species (Norouzian et al., 2006), commercial or industrial glucoamylases with moderate thermostability and high activity are mainly derived from *Aspergillus niger* (Norouzian et al., 2006), *Rhizopus oryzae* (Wang et al., 2020), and *Talaromyces emersonii* (Nielsen et al., 2002) due to the conversion of starch to glucose (Zong et al., 2022). Because the saccharification processes are usually followed by a liquefaction process of starch and are performed at 60°C for 48–72 h, the glucoamylases required in starch industrials have to possess good thermostability and catalytic activities (Lim and Oslan, 2021; Tong et al., 2021). So, searching for a new source of glucoamylase with potentially applicable properties encompassing elevated temperature, extreme pH, high salinity, organic solvents, surfactants, and specificities (substrate and product) is still of considerable importance (Schmidt et al., 2019). Although some novel glucoamylases have been found and characterized for industrial applications (Guo et al., 2019; Karim et al., 2019; Lincoln et al., 2019; Zhang et al., 2019; Wang et al., 2020; Lago et al., 2021; Wayllace et al., 2021), they did not achieve industrially desirable traits.

In addition to exploring novel enzymes with desirable properties in nature, attempts are being made to improve the properties of the existing enzymes by protein engineering techniques to make them suitable for industrial applications (Parashar and Satyanarayana, 2016; Sharma et al., 2019). These techniques mainly include rational design, semi-rational design, directed evolution (error prone PCR and DNA shuffling), and fusion (Schmidt et al., 2019; Sharma et al., 2019; Lim and Oslan, 2021; Tong et al., 2021). However, the design of chimeric enzymes by fusing different domains from native enzymes is considered to be a straightforward method for generating a novel enzyme with improved catalytic properties (Parashar and Satyanarayana, 2016; Ali et al., 2020). Many chimeric enzymes have enhanced thermostability, catalytic efficiency, substrate specificity, and product selectivity (Parashar and Satyanarayana, 2016; Parashar and Satyanarayana, 2017; Peng et al., 2018). For example, Parashar et al. constructed a chimeric α-amylase by fusing the domains of amylases from *Bacillus acidicola* and G*eobacillus thermoleovorans*, which showed enhanced thermostability and catalytic efficiency (Parashar and Satyanarayana, 2016).

In general, the GA from filamentous fungi consists of three regions: the N-terminal catalytic domain (CD), C-terminal starch-binding domain (SBD), and a linker composed primarily of serine and threonine residues between the CD and SBD (Sauer et al., 2000; Guo et al., 2019). Studies have showed that the CD, linker, and SBD sequences of glucoamylase from *A. niger*, respectively, contain residues 1–471, 472–508, and 509–616 (Lee and Paetzel, 2011; Suyama et al., 2017). Of course, a few glucoamylases lack the SBD; however, it has almost the same hydrolytic rate against soluble starch as the average GA, but against insoluble starch, it has a much less hydrolytic rate than the average GA (Sauer et al., 2000; Cornett et al., 2003; Hostinova et al., 2003). The study of the three structural domains of glucoamylase has been carried out, and the results indicated that the positioning of the SBD related to the catalytic domain had an effect on soluble starch/insoluble starch; the effect on soluble starch was much less than that on insoluble starch (Cornett et al., 2003). Moreover, the presence/absence of O-glycosylated linker connecting the CD and SBD of glucoamylase also affected the hydrolysis of insoluble starch (Sauer et al., 2001; Lin et al., 2007).

Domain shuffling is a method to generate chimeric proteins with novel structural and functional properties by fusing domains of different proteins (Cornett et al., 2003; Marin-Navarro et al., 2011; Gomis-Cebolla et al., 2020). In our laboratory, there are two industrial strains, *Aspergillus niger* Ld418A1 and *Talaromyces emersonii* Ld418 TE, producing glucoamylases GAA1 and GATE which are used in saccharification processes of starch. GAA1 has a good thermostability, but its optimal temperature is lower than that of GATE. On the contrary, glucoamylase GATE has a higher optimal temperature, but its thermostability is lower than that of GAA1. We aim to create a chimeric glucoamylase combining their positive characteristics using the domain shuffling method. So, the three domains from the two glucoamylase genes GAA1 and GATE were amplified and then shuffled by overlap PCR to generate six glucoamylase genes: GA1–GA6. The chimeric GA1 and GA2 obtained enhanced thermostability and had almost the same catalytic efficiency as GATE.

## Materials and Methods

### Strains, Plasmids, and Medium

*Aspergillus niger* Ld418A1 and *Talaromyces emersonii* Ld418 TE were used as donors of glucoamylase genes and cultivated in a PDA medium. Cloning was done in *Escherichia coli* DH5α, and *Saccharomyces cerevisiae* W303-1B (Xu et al., 2010) was used as a host strain for the expression of glucoamylase genes, which were grown in an LB medium and a YPD medium, respectively.

The pUCm-T vector was purchased from TaKaRa (Dalian, Liaoning, China), and the pYPGE15 vector (Xu et al., 2010) was provided by East China Normal University (Shanghai, China). The restriction endonucleases and DNA ligases involved in the molecular manipulation were bought from Fermantas (Tianjin, China). The SC-U plate containing 0.67% YNB, 0.115% mixture of basic amino acids, and adenine without uracil, 2% glucose, and 2% agar was used for screening yeast transformants. The YWSX (SC-U without glucose, 1% soluble starch, 0.02% trypan blue) plate was used for testing the expression of transformants.

## Methods

### Cloning Glucoamylase Genes

Filamentous fungi Ld418A1 and Ld418 TE were, respectively, cultivated in 50 ml PDA medium at 28°C for three days. The total RNA was extracted by the TRIzol method using a TRIquick reagent (Solarbio Science and Technology Co., Ltd., Beijing China) and processing as per the instructions. The cDNA was obtained by reverse transcription using RNase inhibitor, dNTP, M-MLV, and the primers ANR TER (Table 1), which were designed and synthesized according to the mRNA sequence of glucoamylase genes from *A. niger* in the NCBI database (GenBank Accession No. BD087377) and *T. emersonii* in the NCBI database (GenBank Accession No. AJ304803.1). The glucoamylase genes GAA1 and GATE were synthesized by PCR using the obtained cDNA as a template.

**TABLE 1:** Sequences of the primers used for this research.

Primer	Primer sequence (5–3′)	Restriction enzyme
ANF	ATG​TCG​TTC​CGA​TCT​CTA​CTC​GCC	
ANR	TCA​CAG​TGT​ACA​TAC​CAG​AGC​GGG	
TEF	ATG​GCG​TCC​CTC​GTT​GCT​GGC	
TER	TCA​CTG​CCA​ACT​ATC​GTC​AAG​AAT​G	
ANWF	CGGAA​TTCATG​TCG​TTC​CGA​TCT​CTA​CTC​GCC	*Eco*R *I*
ANWR	CCGCTC​GAGTCA​CAG​TGA​CAT​ACC​AGA​GCG​GG	*Xho* I
TEWF	GCTCT​AGAATG​GCG​TCC​CTC​GTT​GCT​GGC	*Xba* I
TEWR	CCGCTC​GAGTCA​CTG​CCA​ACT​ATC​GTC​AAG​AAT​G	*Xho* I
GAA1F	GCT​ACC​AAC​ACC​GTC​TGG​CCA​AGC​ATC​GTG​GCT​ACT​GGC​GGC​ACC​ACT​A	
GAA1R	GGT​TGT​TGA​GCT​GCC​AGA​GCC​AGA​ACT​CGG​CCA​CGA​GGT​GAC​AGT​CAC	
GAA2F	GTC​AGC​ACC​AGT​TAC​GGG​GAG​ACA​TGT​ACC​ACT​CCC​ACC​GCC​GTG​GCT​G	
GAA2R	GGG​GAT​CGA​GCC​GGC​CAG​GTA​GAT​GGA​GGT​TGA​TGA​CGT​ACT​GGT​GCT​G	
GATE1F	GTG​ACT​GTC​ACC​TCG​TGG​CCG​AGT​TCT​GGC​TCT​GGC​AGC​TCA​ACA​ACC	
GATE1R	TAG​TGG​TGC​CGC​CAG​TAG​CCA​CGA​TGC​TTG​GCC​AGA​CGG​TGT​TGG​TAG​C	
GATE2F	CAG​CAC​CAG​TAC​GTC​ATC​AAC​CTC​CAT​CTA​CCT​GGC​CGG​CTC​GAT​CCC​C	
GATE2R	CAG​CCA​CGG​CGG​TGG​GAG​TGG​TAC​ATG​TCT​CCC​CGT​AAC​TGG​TGC​TGA​C	

The fragments (CD, SBD, or linker) amplified for each primer pairs: ANF/ANR, GAA1; TEF/TER, GATE; ANWF/ANWR, GAA1; TEWF/TEWR, GATE; ANWF/GAA1R, CD, of GAA1; GAA1F/GAA2R, linker of GAA1; GAA2F/ANWR, SBD, of GAA1; TEWF/GATE1R, CD, of GATE; GATE1F/GATE2R, linker of GATE; GATE2F/TEWR, SBD, of GATE.

### Domain Shuffling

**FIGURE 1:**
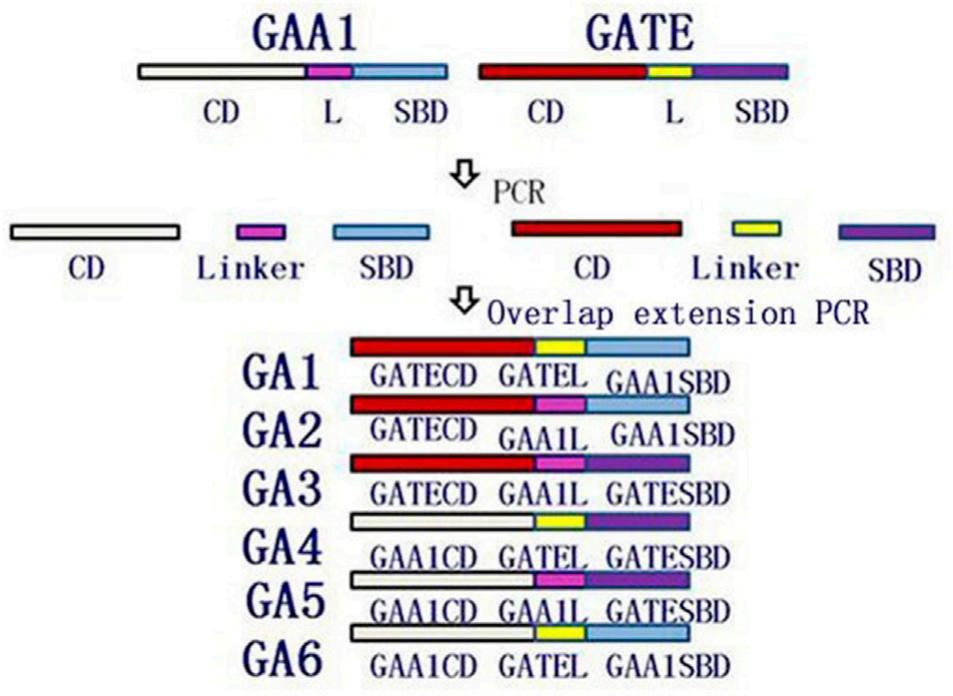
Schematic of creating recombinant glucoamylase genes GA1–GA6 by domain shuffling using the overlap extension PCR. CD, linker, and SBD fragments from GAA1 and GATE genes were, respectively, amplified by PCR and were used as templates of the overlap extension PCR to amplify GA1–GA6 genes.

The different domains (CD, linker, and SBD) were amplified by PCR using glucoamylase genes GAA1 and GATE as templates and corresponding primers, which are listed in Table 1. Domain shuffling was conducted, as shown in Figure 1, using the overlap extension PCR. For example, recombinant gene *GA1* was amplified by overlap extension PCR using CD and linker fragments from GATE and SBD fragments from GAA1 as templates and TEWF/ANWR as primes. Six recombinant genes GA1–GA6 were generated similar to GA1 by overlap extension PCR.

The overlap extension PCR was carried out under the following conditions: the first step was initial denaturation at 95°C for five min, followed by six cycles of 95°C for 45 s, 72°C for three min, six cycles of 95°C for 45 s, 55°C for 45 s, and 72°C for four min, 20 cycles at 95°C for 45 s, 60°C for 45 s, and 72°C for four min, and the ﬁnal extension was carried out at 72°C for ten min.

### Construction and Transformation of Recombinant Expression Vectors

The genes GAA1, GATE, and GA1–GA6 were, respectively, ligated into pYPGE15 and transformed into *E. coli* DH5α by CaCl_2_ transformation. The recombinant expression vectors were identified by the digestion of restriction enzymes and sequencing by Shenggong Biotech Company. The *S. cerevisiae* W303-1B was transformed with the constructed vectors pYPGE15-GAA1, pYPGE15-GATE, and pYPGE15-GA1–pYPGE15*-*GA6 by using the electroporation method and was then inoculated into the SC-U plate for screening transformants.

### Expression, Activity Assay, and Purification of GAs

The monoclonal transformants on the SC-U plate were inoculated into the YWSX plate and incubated for 60 h at 30°C in order to screen the high-level GA expression strain by determination of the ratio (DH/DC) of the hydrolysis circle diameter (DH) and colony diameter (DC). The engineered strains with higher ratios were inoculated into shake flasks and fermented for three days at 30°C for the activity assay and purification of GAs.

The GA activity in the fermentation supernatant was determined according to a modified method (Miller, 1959). With this method, 25 ml soluble starch (20 g/L) was mixed with 5 ml 0.05 mol/L sodium acetate buffer (pH 4.5) and then incubated at 40°C for five min in a tube. Furthermore, 2 ml standard diluted enzyme solution was added into the tube and incubated at 40°C for 30 min. Then, 0.2 ml NaOH (200 g/L) was added. Finally, the diluted solution (diluted ten times) and 1.5 ml DNS (3, 5-dinitrosalicylic acid) was mixed in the tube and immediately transferred to boiling water for five min to end the reaction. The absorbance of these mixtures was measured at a wavelength of 520 nm with sterile water as the control. One unit (U) of the enzyme activity was defined as the amount that catalyzes soluble starch and produces 1 µmol glucose per minute at 40°C, pH 4.5.

The purification of the GAs was performed as previously reported with minor modifications (Cornett et al., 2003). The fermentation supernatant collected by centrifugation was filtered through Φ0.45-μm filter membrane and further concentrated through a 10-kDa cutoff Amicon ultrafiltration system, followed by diafiltration with three times its wash/diafiltration buffer (0.1 mol/L sodium acetate, pH 4.3/1.5 mol/L NaCl). The crude GAs were further purified through acarbose affinity chromatography. The purity of the recombinant GAs was examined by SDS-PAGE.

### Effects of Temperature on the GA Activity and Stability

The optimum temperature of the GAs was evaluated by measuring the enzyme activity at different temperatures (35–80°C) at pH 4.5. The effect of temperature on enzyme stability was determined by measuring the residual activity after being kept at different temperatures (45–80°C) for six h and cooled down rapidly on ice, and the residual enzyme activity of the recombinant GAs was individually measured at pH 4.5. A stability curve of temperature was plotted when the enzyme activity at optimal temperature was set as 100%.

### Effects of pH on the GA Activity and Stability

The optimum pH of the GA was measured by varying the pH of the reaction buffer using 0.05 mol/L sodium acetate buffer at desired pH (from 3.0 to 6.5) at 40°C. In order to test the stability of GA in different pH, the GA solution was adjusted to the desired pH (3.0–6.5) using acetic acid and was kept at 4°C for 6 h, and then the residual enzyme activity of the recombinant GAs was individually measured at 40°C. A stability curve of pH was plotted when the uninsulated enzyme activity at the optimal pH was set as 100%.

### Determination of Kinetic Parameters

Kinetic studies were performed in 0.05 mol/L sodium acetate at 40 °C, pH 4.5 using soluble starch as the substrate with concentrations ranging from 0.125 km to 8 km. The *K*m and *K*cat values of the purified GAs were calculated using the Lineweaver–Burk plot method.

### Structural Analysis of Chimeric Glucoamylases GA1 and GA2

The homologous analysis of DNA and the amino acid sequence alignment of recombinant GAs were conducted using the tool DNAMAN. The tool PSIPRED (Jones, 1999) was used to predict the secondary structure of recombinant GAs. The tertiary structure of recombinant glucoamylases was predicted by SWISS-MODEL (Arnold et al., 2006). All pieces of software were used to make a comparison of the structure between GAA1 and GATE, and GA1 and GA2, and the change of structure was also discussed.

## Results

### Cloning GAA1 and GATE and Rearranging Domains

The glucoamylase genes GAA1 and GATE were, respectively, cloned and sequenced. According to the results, the size of genes GAA1 and GATE were 1776 bp and 1857 bp, respectively, consistent with the reported sequences. As shown in Figure 1, CD, linker, and SBD regions of genes GAA1 and GATE were, respectively, amplified by PCR. Then, six recombinant genes GA1 (GATECD + GATEL + GAA1SBD), GA2 (GATECD + GAA1L + GAA1SBD), GA3 (GATECD + GAA1L + GATESBD), GA4 (GAA1CD + GATEL + GATESBD), GA5 (GAA1CD + GAA1L + GATESBD), and GA6 (GAA1CD + GATE1L + GAA1SBD) were obtained by overlap extension PCR and further identified by sequencing; the lengths of these were GA1 as 1776 bp, GA2 as 1791 bp, GA3 as 1872 bp, GA4 as 1857 bp, GA5 as 1842 bp, and GA6 as 1761 bp.

### Construction and Screening of Engineered Strains

**FIGURE 2:**
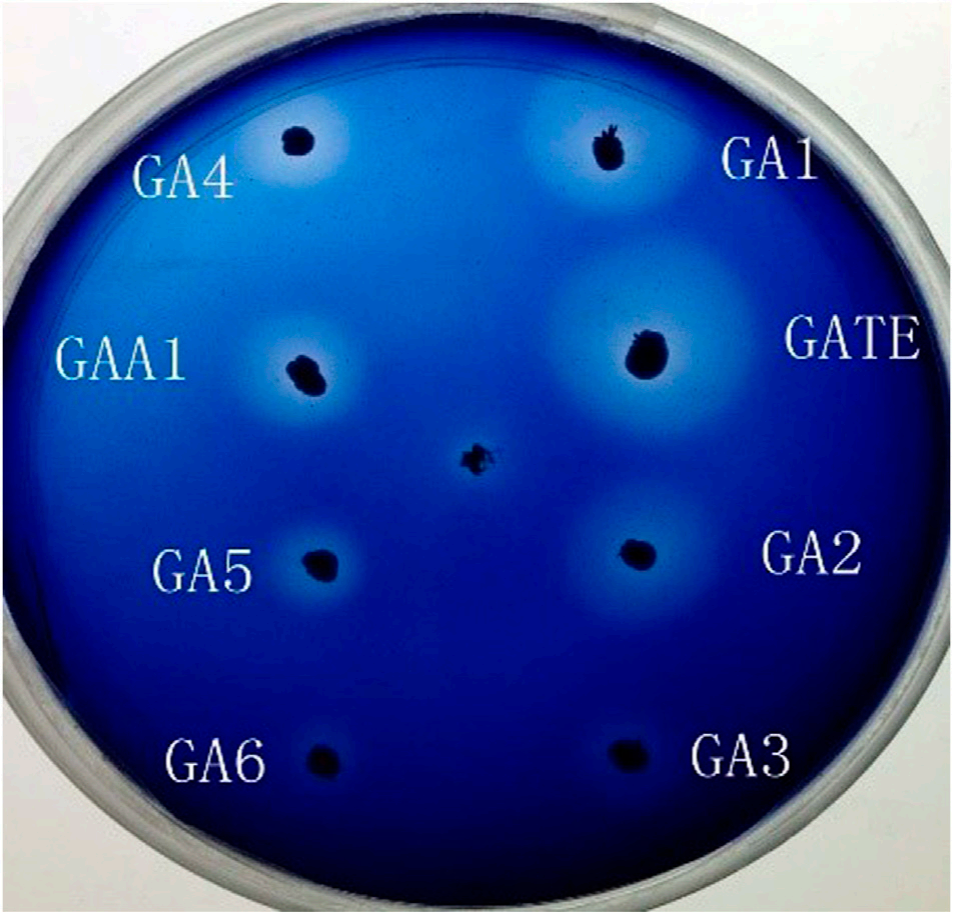
Transparent hydrolysis circle test of the recombinant strains expressing chimeric glucoamylases GAA1, GATE, GA1, GA2, GA3, GA4, GA5, and GA6, respectively.

The recombinant expression vectors pYPGE15-GA1, pYPGE15-GA2, pYPGE15-GA3, pYPGE15-GA4, pYPGE15-GA5, and pYPGE15-GA6; the control vectors pYPGE15-GAA1 and pYPGE15-GATE were constructed and transformed into *S. cerevisiae* W303-1B; and the possible positive transformants were selected from the SC-U plate and inoculated to the YWSX plate to screen the recombinant strains which could express highl-level GAs. The engineered strains YGAA1, YGATE, YGA1, YGA2, YGA3, YGA4, YGA5, and YGA6 could produce transparent hydrolysis circles around their colonies on the YWSX plate, and it is found by calculating the ratio of the diameters of hydrolysis circles and colonies, and the ratios produced by the engineered strains YGAA1, YGATE, YGA1, and YGA2 were obviously higher than those produced by the other engineered strains (Figure 2). It is generally believed that the higher the ratio was, the stronger the ability that strains produce GAs will be. So, the three strains with the highest ratio in each class of engineered strains were inoculated into flasks for fermentation, and GA activities in the fermentation broth were determined.

### Enzymatic Activity Assay and SDS-PAGE of Recombinant GAs

**FIGURE 3:**
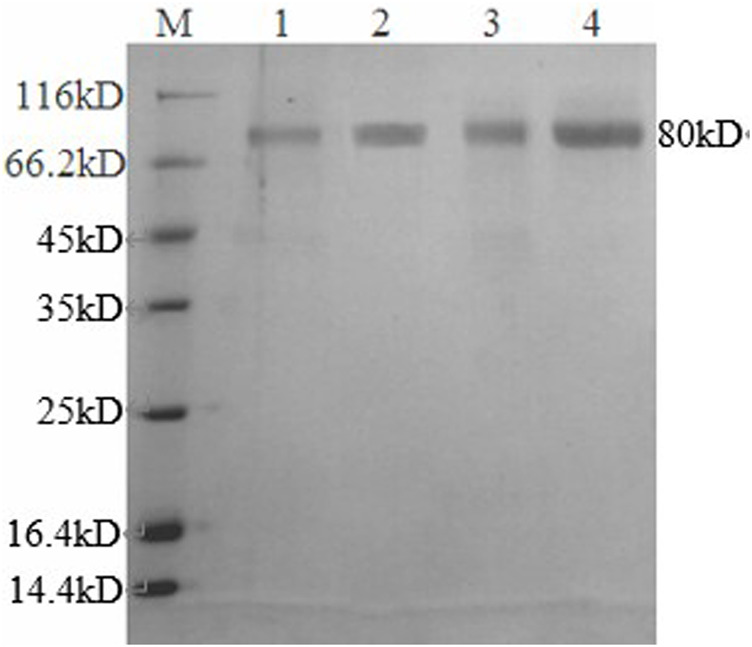
SDS-PAGE of recombinant GAs. M: protein marker; 1: GAA1, 2: GA1; 3: GA2; 4: GATE.

The enzyme activity was determined, and GATE had the highest enzyme activity (73.4 IU/ml), followed by the recombinant GA1 (62.8 IU/ml), GA2 (31.6 IU/ml), and the original GAA1 (27.6 IU/ml), and the activities of the other four recombinants GA3*,* GA4*,* GA5, and GA6 were, respectively, 6.1, 21.1, 16.7, and 19.7 IU/ml, which had even lower enzyme activity than GAA1. It showed that domain shuffling could affect the expression and activity of glucoamylase. So, GA1 and GA2 with higher activity than the original GAA1 were purified for the analysis of their enzymatic properties. The SDS-PAGE result indicated that apparent molecular weights of the recombinant GAs were about 80 kDa (Figure 3), which were all larger than their theoretical molecular weights. It is possible that the recombinant GAs were glycosylated due to presence of O-glycosylated linker. The result also showed that their purity reached electrophoretic purity.

### Analysis of Enzymatic Properties of Recombinant Glucoamylases GA1 and GA2

#### Optimal Temperature and Thermal Stability

**FIGURE 4:**
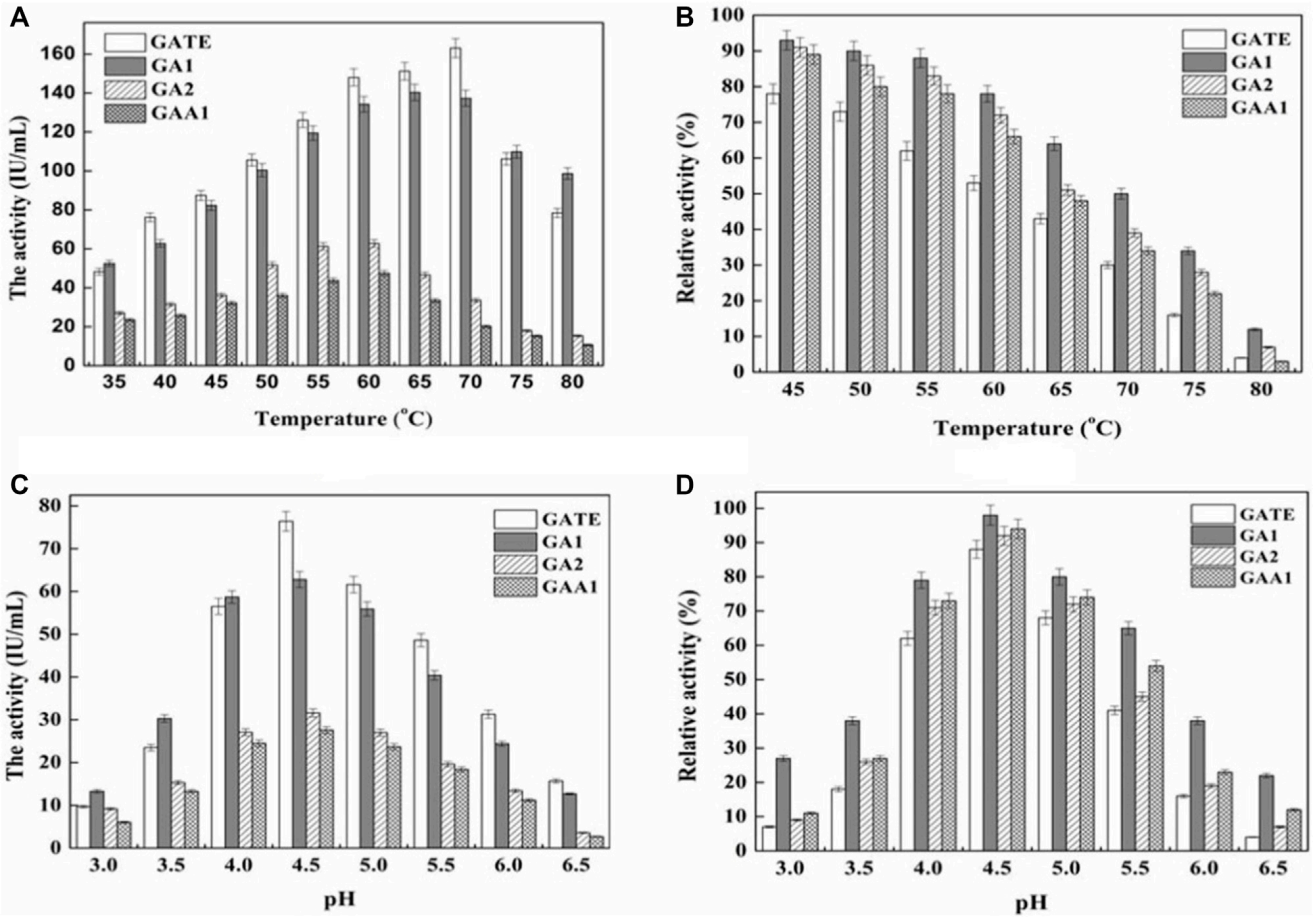
Effect of temperature and pH on activities and stabilities of recombinant GAs. **(A)** Effect of temperature on the activity of the GA. GA activity was measured at various temperatures (35–80°C) at pH 4.5. **(B)** Thermal stability of the purified GA. After being kept at different temperatures (45–80°C) for six h and cooled down rapidly on ice, the residual enzyme activity of GA was measured at pH 4.5. The enzyme activity at optimal temperature was set as 100%. At 50°C, the relative activity of GATE, GA1, GA2, and GAA1 was 73, 90, 86, and 80%, respectively, corresponding to 100%. At 60°C, the relative activity of GATE, GA1, GA2, and GAA1 was 53, 78, 72, and 66%, respectively, corresponding to 100%. **(C)** Effect of pH on GA activity. GA activity was measured in the pH range of 3.0–6.5 at 40°C. The maximum activity was obtained at pH 4.5. **(D)** pH stability of the GA. GA activity was determined after being kept at various pH values (3.0–6.5) for 6 h at 40°C. A stability curve of pH was plotted when the enzyme activity at optimal pH was set as 100%. The relative activity of GA1, GATE, GAA1, and GA2 was 98, 88, 94, and 92%, respectively.

The results (Figure 4A) showed that the optimal temperature of GATE, GA1, GA2, and GAA1 was 70°C, 65°C, 60°C, and 60°C, respectively. Under the optimum temperature of GATE, GA1, GA2, and GAA1, the enzyme activities of the fermentation broth were 163.08 IU/ml, 140.36 IU/ml, 62.81 IU/ml, and 47.53 IU/ml, respectively. The results (Figure 4B) showed that the activity of recombinant GA declined after being preserved at different temperatures for six h. The activity of GATE, GA1, GA2, and GAA1 decreased to 73, 90, 86, and 80% at 50°C, and 53, 78, 72, and 66% at 60°C, respectively. The results revealed that the activities of recombinant GATE and GAA1 decreased much faster than those of recombinant GA1 and GA2, where the thermostability of GA1 was the highest, followed by GA2. Both were higher than the original GAA1 and GATE, indicating that the thermostability of GA could be enhanced by domain shuffling.

#### The Optimum pH and pH Stability

According to Figure 4C, the optimum pH of the original GAA1 and GATE and the recombinant GA1 and GA2 was pH 4.5. Under the optimum pH, the activity of GATE was the highest, followed by GA1, and the activity of GA2 was higher than that of GAA1. Figure 4D illustrates that the relative activity of GA1, preserved at pH 4.5, 40°C for six h, was still 98%, which was higher than that of GATE, GAA1, and GA2 (88, 94, and 92%, respectively). The activity of the recombinant GAs was decreased when the pH was far away from the optimum pH. However, the reduced rate of GA1 was slower than that of others, indicating that the pH stability of GA1 was higher than that of GAA1, GA2, and GATE.

#### Determination of Kinetic Parameters

The kinetic parameters of the recombinant GAs are shown in Table 2. The *Km* values of GATE, GA1, GA2, and GAA1 were 32.58 s^−1^, 30.01 s^−1^, 26.29 s-^1^, and 18.71 s^−1^, respectively, showing that the affinity of GA1 to the substrates was similar to that of GATE; however, the affinity of GA2 to substrates was lower than that of GATE but superior to the affinity of GAA1 to substrates. By the analysis of their *Kcat* values, it was found that *Kcat* values of GA1 (0.15 mg/ml) and GA2 (0.15 mg/ml) were very similar to those of GATE (0.16 mg/ml) but outclassed GAA1 (0.12 mg/ml), indicating that *Kcat* values may be related to the SBD of glucoamylases. As for *Kcat*/*Km*, *Kcat*/*Km* values of GATE, GA1, GA2, and GAA1 were, respectively, 203.62 mg/mL/s, 200.07 mg/mL/s, 175.27 mg/mL/s, and 155.92 mg/mL/s, indicating that GATE and GA1 had almost the same catalytic efficiency. Nevertheless, the catalytic efficiency of GA2 was less than that of GATE but higher than that of GAA1. These results revealed that domain shuffling could still influence catalytic efficiency.

**TABLE 2:** Kinetic parameters of recombinant GAs.

Enzyme	*K*cat (/s)	*K*m (mg/ml)	*K*cat/*K*m (mg/mL/s)
GAAI	18.71 ± 1.81	0.12 ± 0.02	155.92 ± 8.91
GATE	32.58 ± 1.52	0.16 ± 0.03	203.62 ± 6.28
GA1	30.01 ± 1.22	0.15 ± 0.06	200.07 ± 7.68
GA2	26.29 ± 1.38	0.15 ± 0.05	175.27 ± 5.98

### Structural Analysis of Chimeric Glucoamylases GA1 and GA2

#### Analysis of the Primary Structure

Through the similarity comparison of amino acid sequences between recombinant GAs and original GAs, GAA1 and GATE had a similarity of 55.11%, GATE and GA1, GA2 had a similarity of 86.88 and 84.19%, respectively, GA1 and GA2 was 94.30%. Marín-Navarro (Marin-Navarro et al., 2011) indicated that domain shuffling of different enzymes might evolve new enzymes and improve the enzymatic properties due to possible changes in the secondary and tertiary structures of the enzymes.

#### Prediction of the Secondary Structure

**FIGURE 5:**
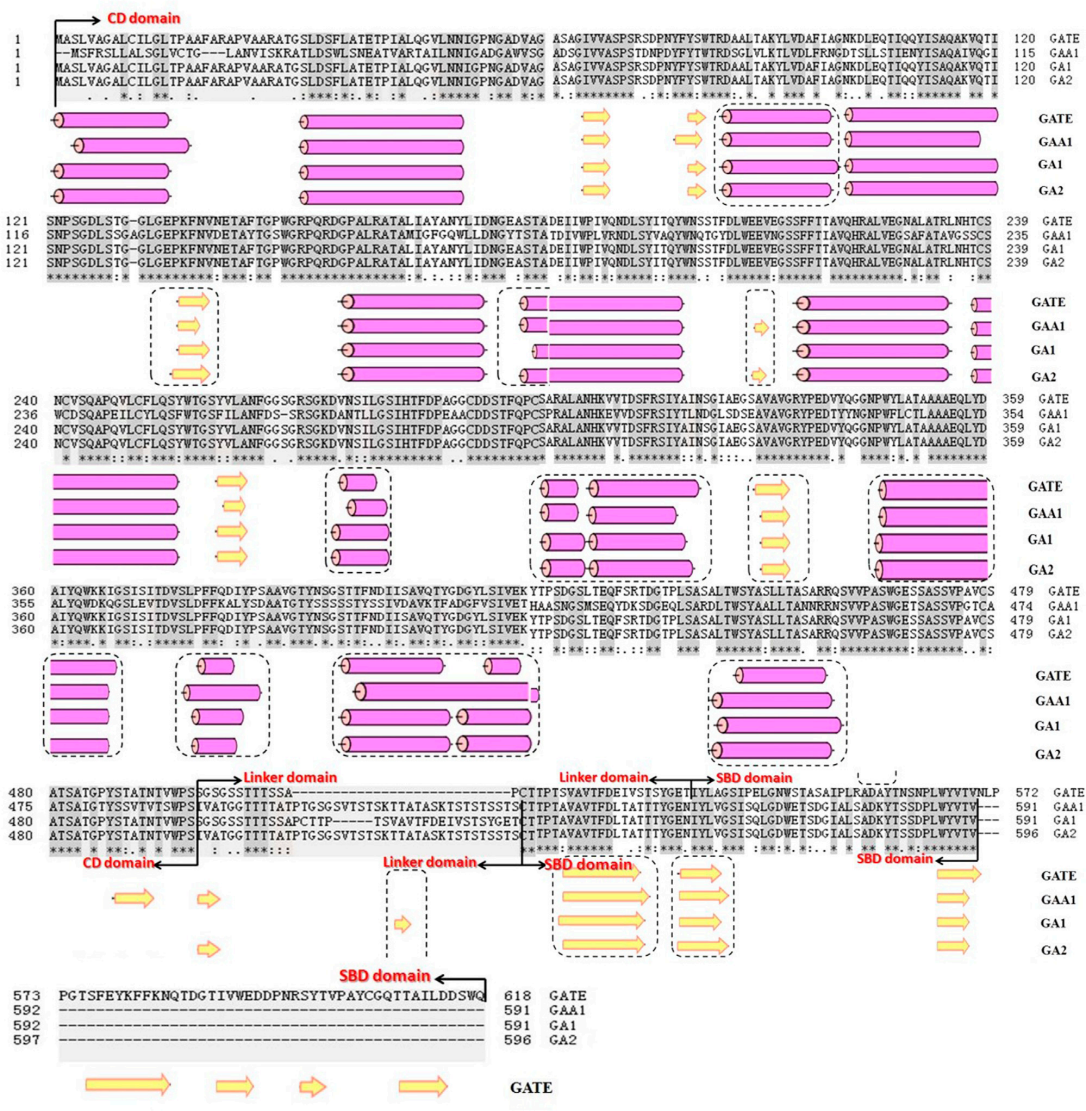
Prediction and comparison of secondary structures of GA1, GA2, GATE, and GAA1 using the tool PSIPRED. The yellow arrow means β-sheet, and the circular column means α-helix.

According to the analysis result of PSIPRED (Jones, 1999) (Figure 5), the number of α-helixes of GA1 and GA2 did not change, but the length of some α-helixes changed. Compared with GATE, the length of α-helixes at seven regions (98, 273–281, 300–305, 344, 378–384, 396–420, and 433–460, amino acid number) increased and extended, especially at the regions (396–420 and 433–460). The longer the α-helix is, the higher the thermal stability will be (Stoffer et al., 1993). Figure 5 also shows that both the number and length of the β-sheet of GA1 and GA2 changed compared with GATE. GA1 had nine β-sheets, which was three β-sheets less than GATE, and formed one new β-sheet (516–518 region), which did not exist in GATE and GAA1. The length of the β-sheet of GA1 increased at the 514–524 regions and decreased at 328–332 and 565–569 regions, which were the same as those of GAA1. However, GA2 had ten β-sheets, which was two β-sheets less than GATE, and formed two new β-sheets (200–209 and 516–518 regions) which only existed in GAA1. The length of the β-sheet of GA1 increased at the 134–139 and 530–537 regions and decreased at the 328–332 and 565–569 regions, which were consistent with GAA1. These differences might cause GA1 and GA2 to have a more stable structure than GATE and GAA1. The 179 and 400 sites of amino acids in GA from *A. niger* have been identified as the catalytic sites (Frandsen et al., 1994; Sierks et al., 1990; Svensson et al., 1990), so a change in the second structure near these catalytic sites might affect their activities. GA1, GA2, and GATE shared the same CD sequences only because their linker and/or SBD sequences were different. Their second structures in the CD showed significant differences. Moreover, there was an increasing frequency at the C-terminal near the CD domain, which indicated that the linker and SBD near the CD domain might influence the secondary structure of the CD domain. The linker and SBD of GATE contained more numbers of β-sheets than those of GAA1, GA1, and GA2. These β-sheets might influence the secondary structure of GATE’CD domain and strengthen the binding of the enzyme with the substrate (Coutinho and Reilly, 1994a). Jia et al. (2004) indicated that there was no complete correspondence between the amino acid sequence and conformation of the protein. Luo and Dong (1988) also showed that the amino acids near the CD domain played a decisive role in the formation of the secondary structure, which mainly had effects on the specificity and catalytic activity of enzymes. The structural differences of the α-helix and β-sheet between chimeric GA1, GA2, and the original GATE and GAA1 contributed to knowing the higher structure of GA and the action mechanism of GA to the substrate (Coutinho and Reilly, 1994b).

#### Prediction of the Tertiary Structure

**FIGURE 6:**
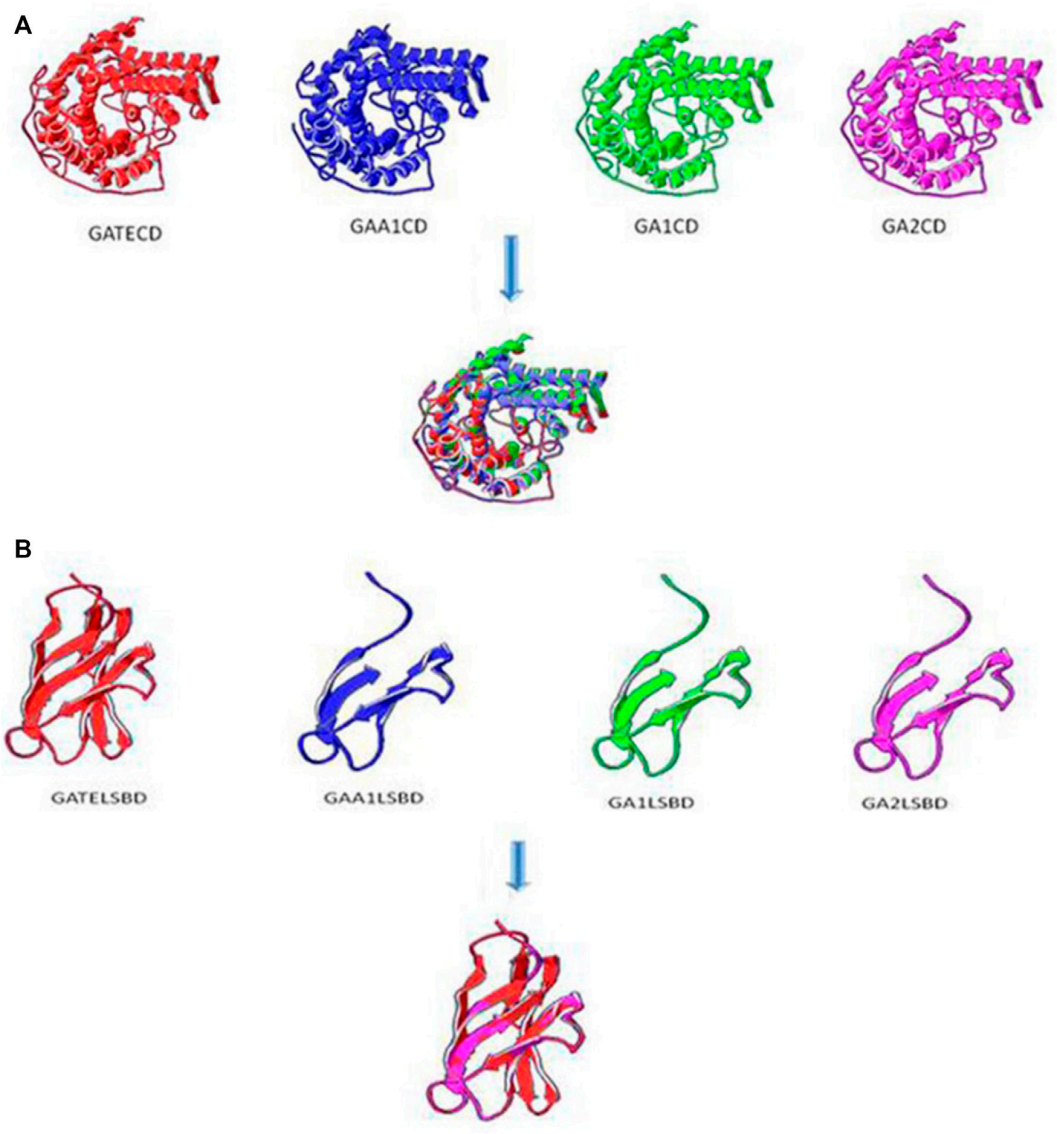
Tertiary structure prediction of CD and SBD. **(A)** Tertiary structure prediction of CD domains. The homology modeling template of GAA1CD had a high-degree homology to PDB: 3eqaA protein and GATECD, GA1CD, and GA2CD were non-high-degree homology to PDB: lgaiA protein. **(B)** Tertiary structure prediction of SBD domains with Linker. The homology modeling template of GAA1SBD, GATESBD, GA1SBD, and GA2SBD were all PDB: 11acoA protein.

The tertiary structure of chimeric glucoamylases was predicted by SWISS-MODEL (Arnold et al., 2006). The CD domains are described in Figure 6A, and the three-dimensional conformation of GATECD, GAA1CD, GA1CD, and GA2CD had a similarity. The homology modeling template of GAA1CD had a high-degree homology to the PDB: 3eqaA protein and GATECD, GA1′CD, and GA2CD had a non-high-degree homology to the PDB: lgaiA protein. As Figure 6A shows, the C-terminal α-helix structure sited on the outside of the catalytic activity between GATE and GAA1 was different, whereas that of GA1, GA2, and GATE was alike. The GA2 had a β-sheet near the center of the CD domain compared with GA1 and GATE and had no difference with GAA1 in the same domain, which might have influenced the activity of GA2. This was consistent with the prediction of the secondary structure.

The tertiary structures of the SBD domain are shown in Figure 6B. The homology modeling templates of GAA1SBD, GATESBD, GA1SBD, and GA2SBD were all PDB: 11acoA protein. However, there was a high-degree homology on the SBD domain between GAA1, GA1, GA2, and the template but only a certain homology between GATE and the template.

## Discussion

Recombination of the existing protein domain is a straightforward method for the creation of new proteins and obtainment of some positive trait strains (Marin-Navarro et al., 2011; Parashar and Satyanarayana, 2016). This study used two chimeric glucoamylases, GA1 and GA2, with higher thermostability and similar catalytic efficiency. As for the enzyme activities, GATE, GA1, GA2, and GAA1 were 163.08 IU/ml, 140.36 IU/ml, 62.81 IU/ml, and 47.53 IU/ml, respectively. According to the results, GA1, GA2, and GA3 shared the same CD as GATE, while GA4, GA5, and GA6 shared the same CD as GAA1, but the enzyme activity of the former, except for GA3, was much higher than the latter. This demonstrated that the CD was a key factor for determining the enzyme activity. The difference between the recombinant GA1 and original GATE was the application of the SBD of GAA1, and the enzyme activity was approximate (only decreased 14%), showing that the change in the SBD had a slight influence on enzyme activity, which agreed with an earlier study (Cornett et al., 2003). The difference in the enzyme activity between GA1 and GA2 was caused by the different application of linker. The recombinant GA3 shared the same CD and SBD with the original GATE and the same Linker with the original GAA1, but its enzyme activity dropped significantly (decreased 92%), which further indicated the influence of linker on the change of the enzyme activity. The reason for this may be that different linkers changed the structure of the CD and SBD. Some studies have shown that the CD and SBD of fungal GAs are functionally independent, the connective function of the linker is not dependent on a special sequence, and different O-glucosylation patterns of linker influence the stability and secretion of GAs and digestion of raw starch (Coutinho and Reilly, 1994a). So, we speculated that linker affected the activities of chimeric GAs by changing the structure of the CD and SBD, which was consistent with the structural predict of recombinant GAs (Figures 5, 6).

Above all, although each domain of glucoamylase could affect the enzyme activity more or less, the influence of the CD on the enzyme activity was the greatest, followed by linker, and the influence of the SBD on the enzyme activity was weak.

The properties of the recombinant GA1 and GA2 and the original GATE and GAA1 were further analyzed. The optimal temperature of chimeric GA1 was 65°C, which was lower than that of GATE (70°C) but higher than that of GAA1 (60°C), and the optimal temperature of recombinant GA2 was the same as GAA1, but the thermostability of GA1 and GA2 was higher than that of the original GAs, suggesting the domain shuffling of GAs could change the thermal stability, although with a slight decrease in the enzyme activity. The reason for the higher stability of GA1 and GA2, according to the analysis of the secondary structure, may be the increased and extended α-helix of GA1 and GA2, especially at the 396–420 and 433–460 regions; the longer α-helix is, the higher the thermostability will be (Stoffer et al., 1993). Through the analysis of the tertiary structures, it could be seen that the SBD of GATE had four additional structures constructed by the β-sheet. This complex structure might make GATE easier to bind with substrates.

## Conclusion

This study has successfully obtained six chimeric glucoamylase genes by domain shuffling of two glucoamylase genes with different enzymatic properties using overlap extension PCR. Of the six chimeric glucoamylases, only GA1 and GA2 revealed higher enzyme activities than the original GAA1 in the fermentation broth. Meanwhile, GA1 and GA2 also showed enhanced thermostability. Moreover, GA1 had the same catalytic efficiency as GATE. GA2 was slightly less than GATE but still higher than GAA1 in terms of catalytic efficiency. The prediction of the secondary and tertiary structures indicated that the increased and extended α-helix of GA1 and GA2, especially in the poor thermal stability and easily being broken region of the amino acid between 443 and 444, may lead to higher thermostability. In a word, by domain shuffling, two novel thermostability chimeric glucoamylases were created, which offered a feasibility to generate novel enzymes with enhanced properties.

## Data Availability

The original contributions presented in the study are included in the article/Supplementary Material, further inquiries can be directed to the corresponding authors.

## References

[B1] Ali M. Ishqi H. M. Husain Q. (2020). Enzyme Engineering: Reshaping the Biocatalytic Functions. Biotechnol. Bioeng. 117, 1877–1894. 10.1002/bit.27329 32159220

[B2] Arnold K. Bordoli L. Kopp J. Schwede T. (2006). The Swiss-Model Workspace: A Web-Based Environment for Protein Structure Homology Modelling. Bioinformatics 22, 195–201. 10.1093/bioinformatics/bti770 16301204

[B3] Cornett C. A. G. Fang T.-Y. Reilly P. J. Ford C. (2003). Starch-binding Domain Shuffling in *Aspergillus niger* Glucoamylase. Protein Eng. Des. Selection 16, 521–529. 10.1093/protein/gzg066 12915730

[B4] Coutinho P. M. Reilly P. J. (1994a). Structural Similarities in Glucoamylases by Hydrophobic Cluster Analysis. Protein Eng. Des. Sel 7, 749–760. 10.1093/protein/7.6.749 7937705

[B5] Coutinho P. M. Reilly P. J. (1994b). Structure-function Relationships in the Catalytic and Starch Binding Domains of Glucoamylase. Protein Eng. Des. Sel 7, 393–400. 10.1093/protein/7.3.393 8177888

[B6] Frandsen T. P. Dupont C. Lehmbeck J. Stoffer B. Sierks M. R. Honzatko R. B. (1994). Site-Directed Mutagenesis of the Catalytic Base Glutamic Acid 400 in Glucoamylase from *Aspergillus niger* and of Tyrosine 48 and Glutamine 401, Both Hydrogen-Bonded to the .gamma.-Carboxylate Group of Glutamic Acid 400. Biochemistry 33, 13808–13816. 10.1021/bi00250a035 7947792

[B7] Gomis-Cebolla J. Ferreira Dos Santos R. Wang Y. Caballero J. Caballero P. He K. (2020). Domain Shuffling between Vip3aa and Vip3ca: Chimera Stability and Insecticidal Activity against European, American, African, and Asian Pests. Toxins 12, 99. 10.3390/toxins12020099 PMC7076965 32033215

[B8] Guo Y. Tu T. Qiu J. Tong L. Luo H. Yao B. (2019). Characterization and Structure of a Novel Thermostable Glucoamylase from *Talaromyces leycettanus* Jcm12802. Sheng Wu Gong Cheng Xue Bao 35, 616–625. 10.13345/j.cjb.180330 31001948

[B9] Hostinová E. Solovicová A. Dvorský R. Gašperík J. (2003). Molecular Cloning and 3D Structure Prediction of the First Raw-Starch-Degrading Glucoamylase without a Separate Starch-Binding Domain. Arch. Biochem. Biophys. 411, 189–195. 10.1016/s0003-9861(03)00003-1 12623067

[B10] Jia M. Luo L. Liu c. (2004). The Relationship between Protein Secondary Structure and Messenger RNA Secondary Structure. Acta Scientiarum Naturalium Universitatis NeiMongol 35, 55–59.

[B11] Jones D. T. (1999). Protein Secondary Structure Prediction Based on Position-specific Scoring Matrices. J. Mol. Biol. 292, 195–202. 10.1006/jmbi.1999.3091 10493868

[B12] Karim K. M. R. Husaini A. Sing N. N. Tasnim T. Mohd Sinang F. Hussain H. (2019). Characterization and Expression in *Pichia pastoris* of a Raw Starch Degrading Glucoamylase (ga2) Derived from *Aspergillus flavus* Nsh9. Protein Expr. Purif. 164, 105462. 10.1016/j.pep.2019.105462 31351992

[B13] Kumar P. Satyanarayana T. (2009). Microbial Glucoamylases: Characteristics and Applications. Crit. Rev. Biotechnol. 29, 225–255. 10.1080/07388550903136076 19708824

[B14] Lago M. C. Santos F. C. Bueno P. S. A. Oliveira M. A. S. Barbosa‐Tessmann I. P. (2021). The Glucoamylase from *Aspergillus wentii*: Purification and Characterization. J. Basic Microbiol. 61, 443–458. 10.1002/jobm.202000595 33783000

[B15] Lee J. Paetzel M. (2011). Structure of the Catalytic Domain of Glucoamylase from *Aspergillus niger* . Acta Cryst. Sect F 67, 188–192. 10.1107/S1744309110049390 PMC3034606 21301084

[B16] Lim S. J. Oslan S. N. (2021). Native to Designed: Microbial α-amylases for Industrial Applications. PeerJ 9, e11315. 10.7717/peerj.11315 34046253 PMC8139272

[B17] Lin S.-C. Liu W.-T. Liu S.-H. Chou W.-I. Hsiung B.-K. Lin I.-P. (2007). Role of the Linker Region in the Expression of *Rhizopus oryzae* Glucoamylase. BMC Biochem. 8, 9. 10.1186/1471-2091-8-9 17593302 PMC1933424

[B18] Lincoln L. More V. S. More S. S. (2019). Purification and Biochemical Characterization of Extracellular Glucoamylase from *Paenibacillus amylolyticus *strain. J. Basic Microbiol. 59, 375–384. 10.1002/jobm.201800540 30681161

[B19] Luo L. Dong Y. (1988). Statistical Analysis of Peptide Correlation and Prediction of Protein Conformation. Chin. Biochem. J. 4, 173–183. 10.13865/j.cnki.cjbmb.1988.02.012

[B20] Marín-Navarro J. Gurgu L. Alamar S. Polaina J. (2011). Structural and Functional Analysis of Hybrid Enzymes Generated by Domain Shuffling between *Saccharomyces cerevisiae* (Var. Diastaticus) Sta1 Glucoamylase and *Saccharomycopsis fibuligera* Bgl1 β-glucosidase. Appl. Microbiol. Biotechnol. 89, 121–130. 10.1007/s00253-010-2845-3 20821204

[B21] Miller G. L. (1959). Use of Dinitrosalicylic Acid Reagent for Determination of Reducing Sugar. Anal. Chem. 31, 420–428. 10.1021/ac60147a030

[B22] Nielsen B. R. Lehmbeck J. Frandsen T. P. (2002). Cloning, Heterologous Expression, and Enzymatic Characterization of a Thermostable Glucoamylase from *Talaromyces emersonii* . Protein Expr. Purif. 26, 1–8. 10.1016/s1046-5928(02)00505-3 12356463

[B23] Norouzian D. Akbarzadeh A. Scharer J. M. Moo Young M. (2006). Fungal Glucoamylases. Biotechnol. Adv. 24, 80–85. 10.1016/j.biotechadv.2005.06.003 16091302

[B24] Parashar D. Satyanarayana T. (2016). A Chimeric α-amylase Engineered from *Bacillus acidicola* and *Geobacillus thermoleovorans* with Improved Thermostability and Catalytic Efficiency. J. Ind. Microbiol. Biotechnol. 43, 473–484. 10.1007/s10295-015-1721-7 26790418

[B25] Parashar D. Satyanarayana T. (2017). Engineering a Chimeric Acid-Stable α-amylase-glucoamylase (Amy-Glu) for One Step Starch Saccharification. Int. J. Biol. Macromolecules 99, 274–281. 10.1016/j.ijbiomac.2017.02.083 28238910

[B26] Peng H. Li R. Li F. Zhai L. Zhang X. Xiao Y. (2018). Extensive Hydrolysis of Raw rice Starch by a Chimeric α-amylase Engineered with α-amylase (AmyP) and a Starch-Binding Domain from *Cryptococcus* Sp. S-2. Appl. Microbiol. Biotechnol. 102, 743–750. 10.1007/s00253-017-8638-1 29159586

[B27] Sauer J. Christensen T. Frandsen T. P. Mirgorodskaya E. McGuire K. A. Driguez H. (2001). Stability and Function of Interdomain Linker Variants of Glucoamylase 1 from *Aspergillus niger* . Biochemistry 40, 9336–9346. 10.1021/bi010515i 11478902

[B28] Sauer J. Sigurskjold B. W. Christensen U. Frandsen T. P. Mirgorodskaya E. Harrison M. (2000). Glucoamylase: Structure/function Relationships, and Protein Engineering. Biochim. Biophys. Acta (Bba) - Protein Struct. Mol. Enzymol. 1543, 275–293. 10.1016/s0167-4838(00)00232-6 11150611

[B29] Schmidt A. Shvetsov A. Soboleva E. Kil Y. Sergeev V. Surzhik M. (2019). Thermostability Improvement of *Aspergillus awamori* Glucoamylase via Directed Evolution of its Gene Located on Episomal Expression Vector in *Pichia pastoris* Cells. Protein Eng. Des. Sel 32, 251–259. 10.1093/protein/gzz048 31891399

[B30] Sharma A. Gupta G. Ahmad T. Mansoor S. Kaur B. (2019). Enzyme Engineering: Current Trends and Future Perspectives. Food Rev. Int. 37, 121–154. 10.1080/87559129.2019.1695835

[B31] Sierks M. R. Ford C. Reilly P. J. Svensson B. (1990). Catalytic Mechanism of Fungal Glucoamylase as Defined by Mutagenesis of Asp176, Glu179 and Glu180 in the Enzyme from *Aspergillus awamori* . Protein Eng. Des. Sel 3, 193–198. 10.1093/protein/3.3.193 1970434

[B32] Stoffer B. Frandsen T. P. Busk P. K. Schneider P. Svendsen I. Svensson B. (1993). Production, Purification and Characterization of the Catalytic Domain of Glucoamylase from *Aspergillus niger* . Biochem. J. 292 (Pt 1), 197–202. 10.1042/bj2920197 8503847 PMC1134288

[B33] Suyama Y. Muraki N. Kusunoki M. Miyake H. (2017). Crystal Structure of the Starch-Binding Domain of Glucoamylase from *Aspergillus niger* . Acta Cryst. Sect F 73, 550–554. 10.1107/S2053230X17012894 PMC5633921 28994402

[B34] Svensson B. Clarke A. J. Svendesen I. Moller H. (1990). Identification of Carboxylic Acid Residues in Glucoamylase G2 from *Aspergillus niger* that Participate in Catalysis and Substrate Binding. Eur. J. Biochem. 188, 29–38. 10.1111/j.1432-1033.1990.tb15367.x 2108020

[B35] Tong L. Zheng J. Wang X. Wang X. Huang H. Yang H. (2021). Improvement of Thermostability and Catalytic Efficiency of Glucoamylase from *Talaromyces leycettanus* Jcm12802 via Site-Directed Mutagenesis to Enhance Industrial Saccharification Applications. Biotechnol. Biofuels 14, 202. 10.1186/s13068-021-02052-3 34656167 PMC8520190

[B36] Wang C. Yang L. Luo L. Tang S. Wang Q. (2020). Purification and Characterization of Glucoamylase of *Aspergillus oryzae* from Luzhou-Flavour Daqu. Biotechnol. Lett. 42, 2345–2355. 10.1007/s10529-020-02956-4 32623532

[B37] Wayllace N. M. Hedín N. Busi M. V. Gomez-Casati D. F. (2021). Characterization of Sdga, a Cold-Adapted Glucoamylase from *Saccharophagus degradans* . Biotechnol. Rep. 30, e00625. 10.1016/j.btre.2021.e00625 PMC8141877 34041001

[B38] Xu K. Zhang H. Blumwald E. Xia T. (2010). A Novel Plant Vacuolar Na^+^/H^+^ Antiporter Gene Evolved by DNA Shuffling Confers Improved Salt Tolerance in Yeast. J. Biol. Chem. 285, 22999–23006. 10.1074/jbc.M109.073783 20457597 PMC2906293

[B39] Zhang M.-Y. Zhao S. Ning Y.-N. Fu L.-H. Li C.-X. Wang Q. (2019). Identification of an Essential Regulator Controlling the Production of Raw-Starch-Digesting Glucoamylase in *Penicillium oxalicum* . Biotechnol. Biofuels 12, 7. 10.1186/s13068-018-1345-z 30622649 PMC6318894

[B40] Zong X. Wen L. Wang Y. Li L. (2022). Research Progress of Glucoamylase with Industrial Potential. J. Food Biochem.. 10.1111/jfbc.14099 35132641

